# Krein support vector machine classification of antimicrobial peptides

**DOI:** 10.1039/d3dd00004d

**Published:** 2023-02-27

**Authors:** Joseph Redshaw, Darren S. J. Ting, Alex Brown, Jonathan D. Hirst, Thomas Gärtner

**Affiliations:** a School of Chemistry, University of Nottingham, University Park Nottingham NG7 2RD UK jonathan.hirst@nottingham.ac.uk; b Academic Ophthalmology, School of Medicine, University of Nottingham Nottingham NG7 2UH UK; c Artificial Intelligence and Machine Learning, GSK Medicines Research Centre Gunnels Wood Road Stevenage SG1 2NY UK; d Machine Learning Group, TU Wien Informatics Vienna Austria; e Academic Unit of Ophthalmology, Institute of Inflammation and Ageing, University of Birmingham Birmingham UK; f Birmingham and Midland Eye Centre Birmingham UK

## Abstract

Antimicrobial peptides (AMPs) represent a potential solution to the growing problem of antimicrobial resistance, yet their identification through wet-lab experiments is a costly and time-consuming process. Accurate computational predictions would allow rapid *in silico* screening of candidate AMPs, thereby accelerating the discovery process. Kernel methods are a class of machine learning algorithms that utilise a kernel function to transform input data into a new representation. When appropriately normalised, the kernel function can be regarded as a notion of similarity between instances. However, many expressive notions of similarity are not valid kernel functions, meaning they cannot be used with standard kernel methods such as the support-vector machine (SVM). The Kreĭn-SVM represents generalisation of the standard SVM that admits a much larger class of similarity functions. In this study, we propose and develop Kreĭn-SVM models for AMP classification and prediction by employing the Levenshtein distance and local alignment score as sequence similarity functions. Utilising two datasets from the literature, each containing more than 3000 peptides, we train models to predict general antimicrobial activity. Our best models achieve an AUC of 0.967 and 0.863 on the test sets of each respective dataset, outperforming the in-house and literature baselines in both cases. We also curate a dataset of experimentally validated peptides, measured against *Staphylococcus aureus* and *Pseudomonas aeruginosa*, in order to evaluate the applicability of our methodology in predicting microbe-specific activity. In this case, our best models achieve an AUC of 0.982 and 0.891, respectively. Models to predict both general and microbe-specific activities are made available as web applications.

## Abstract 2

We use an indefinite kernel method, the Kreĭn support vector machine, to predict antimicrobial activity of peptides.

## Graphical abstract

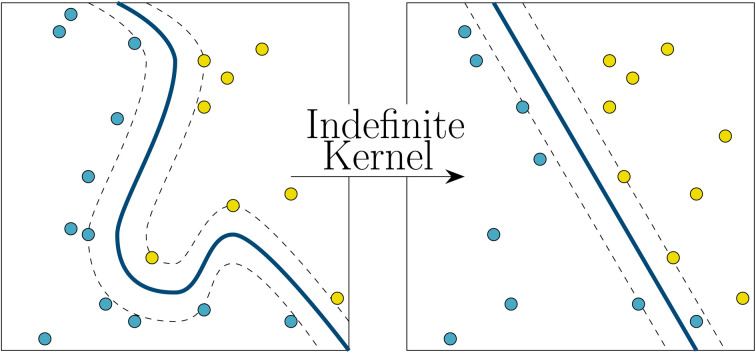

## 1 Introduction

Kernel methods are a class of machine learning algorithms that incorporate a kernel function in order to model non-linear relationships. Standard kernel methods assume that a given kernel function is positive-definite. Those kernel functions which do not satisfy this assumption are known as indefinite kernels. The assumption of positive-definiteness is restrictive, as it limits the number of applicable functions. Recent developments in the theory of learning with indefinite kernels have now removed this requirement, allowing a much broader class of functions to be incorporated into kernel methods.^1–3^ Leveraging these developments, we study the effectiveness of learning with established sequence-similarity functions for the classification of antimicrobial peptides (AMPs) based on their amino acid sequences. We evaluate the ability of the proposed methodology to predict general antimicrobial activity, as well as antimicrobial activity against specific species.

AMPs, also known as host defense peptides, are a class of evolutionary conserved molecules that form an important component in the innate immune system.^4–6^ These molecules are usually made of 12 to 50 amino acid residues, and they typically possess certain properties, including cationicity, 30–50% hydrophobicity, and amphiphilicity. They exhibit good antimicrobial activity against a broad range of bacteria, viruses, fungi, and parasites. In addition, they have an inherent low risk of developing antimicrobial resistance (AMR), largely attributed to their underlying rapid membrane permeabilising activity.^4,7,8^ Such broad-spectrum and rapid antimicrobial activity has prompted researchers to consider AMPs as a potential remedy to the growing problem of AMR, which is a major global health threat.^9,10^ Nonetheless, there has so far been a lack of success in translating AMP-based therapy to clinical use, due to challenges such as complex structure–activity relationship (SAR), drug toxicity, instability in host and infective environment, and low financial incentives.^11,12^ Owing to the complex SAR and the costly and time-consuming process of wet-lab experiments associated with AMP investigations, many researchers have proposed computational approaches, including molecular dynamics (MD) simulations and machine learning (ML) algorithms, to accelerate the discovery and development of potential AMPs for clinical use.^13–19^

Several studies have highlighted the promise of ML algorithms in predicting the antimicrobial activity, dissecting the complex SAR, and informing the drug design of AMPs.^13–15^ A wide range of ML algorithms have been utilised, including random forests,^20^ support vector machines (SVMs)^20–24^ and artificial neural networks.^20–22,25,26^ Many of these algorithms are used in combination with a carefully selected set of peptide features, which can be divided into two categories: compositional and physicochemical. The amino acid composition is the simplest example of a compositional feature, which is a vector containing counts of each amino acid in a given peptide. There are various extensions, such as the reduced amino acid composition^27^ and the pseudo amino acid composition.^28^ When computing the reduced amino acid composition, a peptide is represented in a reduced alphabet in which similar amino acids are grouped together. The pseudo amino acid composition accounts for composition as well as sequence-order information, as this is not considered in the standard amino acid composition. The set of physicochemical features include peptide properties such as the charge, hydrophobicity and isoelectric point.^20,24,29^ These features are typically average values of the respective properties calculated over the length of the peptide.

Classical sequence alignment algorithms, such as the Smith–Waterman^30^ and Needleman–Wunsch^31^ algorithms, are computationally intensive and do not scale well to large problems. Many papers have advocated the use of alignment-free methods to determine sequence similarity.^19,32–35^ The success of these endeavours notwithstanding, sequence alignment functions are effective notions of biological-sequence similarity that can reflect ancestral, structural or functional similarity and therefore should not be overlooked. Several studies have utilised sequence alignment functions for AMP prediction. For example, Wang *et al.*^36^ and Ng *et al.*^37^ utilised the BLAST algorithm^38^ in a classification model by comparing the BLAST similarity scores of a query peptide to all those in the training set. Whilst these approaches led to accurate models, the BLAST algorithm is a heuristic method that finds only approximate optimal alignments. This approximation leads to generally faster results than what could be obtained by the Smith–Waterman algorithm, and it is one of the main reasons practitioners choose to use it. However, on the relatively small datasets in the aforementioned studies, it is interesting to consider whether the same approaches using the optimal alignment score would improve the models.

The SVM is a well-known ML algorithm for classification and can incorporate a kernel function in order to learn non-linear classification boundaries. The kernel function greatly influences the performance of the resulting classification model. When appropriately normalised, a kernel function can be regarded as a similarity function. A useful kernel function should produce similarities that are relevant to the problem. Many expressive notions of similarity are not valid kernel functions,^39–42^ in that they are indefinite, meaning they cannot be used with an SVM. Recent developments have now alleviated this problem, facilitating a much larger class of similarity functions to be used in conjunction with an SVM. Loosli *et al.*^1^ present an algorithm for learning an SVM with indefinite kernels. Their approach relies on a method of stabilisation, meaning there is no guarantee of global optimality. On the other hand, the Kreĭn-SVM^3^ is an algorithm for learning an SVM with indefinite kernels that is guaranteed to find a globally optimal solution.

In this work, we utilised the Kreĭn-SVM algorithm to assess the effectiveness of sequence alignment functions for AMP classification. We performed an empirical comparison of both the local alignment score and Levenshtein distance^43,44^ on two AMP datasets from the literature. Furthermore, we tested the ability of our approach to detect activity against specific species on a dataset of experimentally validated peptides measured against both *Staphylococcus aureus* ATCC 29213 and *Pseudomonas aeruginosa* ATCC 27853, henceforth referred to as *S. aureus* ATCC 29213 and *P. aeruginosa* ATCC 27853. Our trained models are made available as web applications at http://comp.chem.nottingham.ac.uk/KreinAMP, for the prediction of both general and species-specific activities.

## 2 Methods

AMP prediction models were developed using the Kreĭn-SVM algorithm in conjunction with sequence alignment functions, which we formally define in this section. We initially describe the more familiar SVM, before moving on to the Kreĭn-SVM. We then define the Levenshtein distance^44^ and the local alignment score. Finally, we describe our computational and microbiological methodology.

### 2.1 SVM and Kreĭn-SVM

#### 2.1.1 SVM

The SVM is a ML algorithm used for classification. The result of training an SVM is a hyperplane whose distance to the closest training instance, in either class, is maximal. Furthermore, instances from each class are required to reside on separate sides of the hyperplane. New instances are classified based solely on which side of the hyperplane they are located. The distance from the hyperplane to the closest training instance is known as the margin. The hyperplane that maximises the margin is the maximum-margin hyperplane and this is what is produced when training an SVM.

The decision surface associated with a hyperplane is inherently linear, which can be restrictive when the two classes are not linearly separable. This issue is mitigated through the use of a kernel function, which implicitly maps the instances into a new space. The space in which the instances are mapped is known as a reproducing kernel Hilbert space (RKHS), and every kernel function is uniquely associated to a RKHS. When incorporating a kernel function, the SVM finds the maximum-margin hyperplane in the associated RKHS and this can correspond to a non-linear decision surface in the original space. This surface allows greater flexibility when separating the two classes but also increases the possibility of overfitting. Perfectly separating a noisy dataset is often indicative of overfitting. The Soft Margin SVM helps alleviate this problem by allowing some instances to reside on the incorrect side of the hyperplane. Eqn (1) presents the optimisation problem that is solved when training a Soft Margin SVM with L2 loss, commonly known as an L2-SVM. We opted for an L2-SVM as greater penalisation is placed on instances residing on the incorrect side of the hyperplane.1
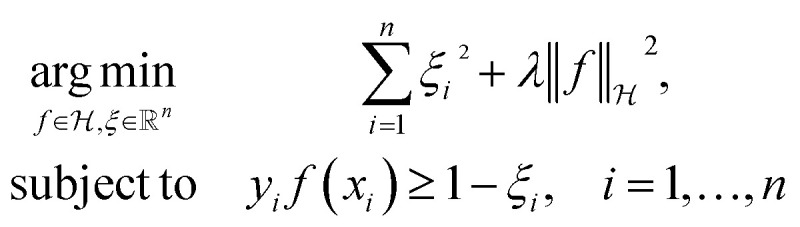
We denote by *x*_*i*_ the *i*-th training instance and by *y*_*i*_ its corresponding label. Furthermore, 
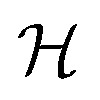
 denotes the RKHS from which a solution is found. The solution is a function 
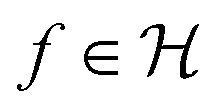
 and a vector of slack variables ξ which minimise the objective function, subject to the constraints. The constraint *y*_*i*_*f*(*x*_*i*_) ≥ 1 − *ξ*_*i*_ imposes that the *i*-th instance lies on the correct side of the hyperplane and that its distance to the hyperplane is greater than or equal to the margin. An instance *x*_*j*_ which does not satisfy this constraint contributes a value of *ξ*_*j*_^2^ to the objective function, where *ξ*_*j*_ is the distance between *x*_*j*_ and the margin. The 
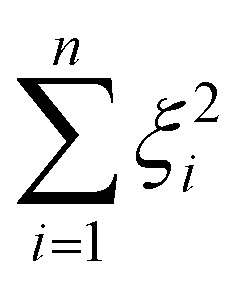
 term in the objective function is the total contribution of all instances which do not satisfy the constraints. The 
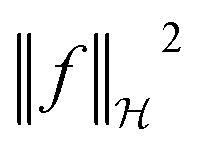
 term is the norm of the considered function and acts as a measure of complexity. The hyperparameter *λ* can be tuned to provide a balance between the number of instances that violate the constraints and the complexity of the considered function.

#### 2.1.2 Kreĭn-SVM

The Kreĭn-SVM is a classification algorithm that is defined to incorporate a much broader class of kernel functions, known as indefinite kernel functions. Similarly to a standard kernel function, an indefinite kernel function implicitly maps instances into a new space. However, the space associated to an indefinite kernel function is known as a reproducing kernel Kreĭn Space (RKKS). Whilst operating in different spaces, the SVM and Kreĭn-SVM are conceptually similar. Both algorithms incorporate a kernel function, find the maximum-margin hyperplane in the associated space and are capable of learning non-linear decision surfaces.2
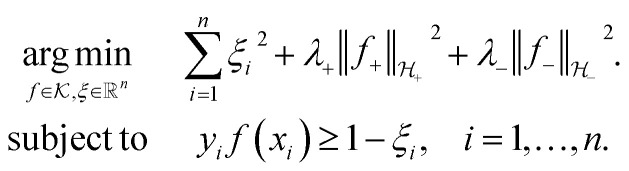
Eqn (2) presents the optimisation problem that is solved when training the Kreĭn-SVM with L2 loss. We denote by 
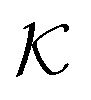
 the associated RKKS. Similarly to the SVM, the solution is a function 
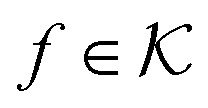
 and a vector of slack variables ξ which minimise the objective function. The first term in the objective function, as well as the constraints, have the same interpretation as in eqn (1). The only notable difference to the SVM is the method of regularisation. Any RKKS 
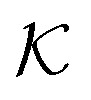
 can be expressed as a direct sum of the form
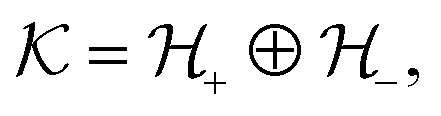
where 
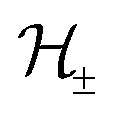
 are RKHSs. This means that any function 
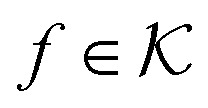
 can be decomposed as *f* = *f*_+_ ⊕ *f*_−_, where 
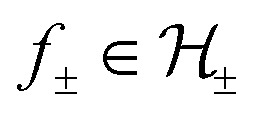
. Hence, regularisation in eqn (2) is performed by separately regularising each component of the decomposition. The hyperparameters *λ*_±_ can be tuned to provide a balance between the number of instances that violate the constraints and the regularisation of each decomposition component.

### 2.2 Sequence similarities and distances

We now proceed to define the sequence similarities used throughout this work. This section closely follows the works of Setubal and Meidanis^45^ and Yujian and Bo.^43^ First, we clarify our terminology.

#### 2.2.1 Notation and terminology

Let Σ be a finite alphabet, Σ^*n*^ ⊆ Σ be the set of all strings of length *n* from Σ and Σ* the set of all strings from that alphabet. A string *s* ∈ Σ* of length *n* is a sequence of characters that can be indexed as *s* = *s*_1_…*s*_*n*_. Given a string *s* ∈ Σ^*n*^, we say that *u* ∈ Σ^*m*^ is a subsequence of *s* if there exists a set of indices *I* = {*i*_1_, …, *i*_*m*_} with 1 ≤ *i*_1_ ≤ … ≤ *i*_*m*_ ≤ *n*, such that *u*_*j*_ = *S*_*i*_*j*__ for *j* = 1, …, *m*. We write *u* = *s*[*I*] for short. We say that *v* ∈ Σ^*l*^ is a substring of *s* if *v* is a subsequence of *s* with index set *J* = {*j*_1_, …, *j*_*l*_} such that *j*_*r*+1_ = *j*_*r*_ + 1 for *r* = 1, …, *l* − 1. That is, *v* is a subsequence consisting of consecutive characters of *s*.

#### 2.2.2 Global alignments

The goal of a sequence alignment is to establish a correspondence between the characters in two sequences. In the context of bioinformatics, a pairwise alignment can indicate ancestral, structural or functional similarities between the pair of sequences. In this section, we provide a formal review of global sequence alignment.

##### Definition 2.1 (global alignment)

Let Σ be an alphabet and let *s* ∈ Σ^*n*^ and *t* ∈ Σ^*m*^ be two strings over Σ. Define Σ_*g*_ = Σ ∪ {“−”} as the extension of Σ with the gap character “−”. The tuple *α*(*s*, *t*) = (*s*′, *t*′) is a global alignment of sequences *s* and *t* if and only if

(1) 
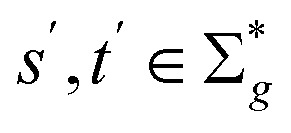


(2) |*s*′| = |*t*′| = *l*, such that max(*n*, *m*) ≤ *l* ≤ *m* + *n*,

(3) The subsequence of *s*′ obtained by removing all gap characters is equal to *s*,

(4) The subsequence of *t*′ obtained by removing all gap characters is equal to *t*,

(5) 
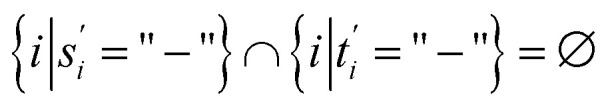
.

Definition 2.1 provides a formal definition of global alignment. Whilst many possible alignments exist for two strings, the goal of sequence alignment is to find an alignment that optimises some criterion. A scoring function, presented in Definition 2.2, can be used to quantify the “appropriateness” of an alignment. An optimal global alignment is then one which is optimal with respect to a given scoring function, as shown in Definition 2.3.

##### Definition 2.2 (scoring functions)

Let Σ be an alphabet, Σ_*g*_ = Σ ∪ {“−”} be the extension of Σ with the gap character “−” and 
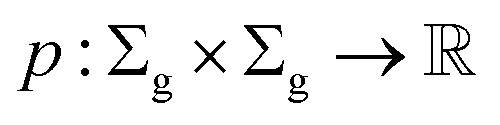
 be a function defined over the elements of Σ_*g*_. We say *p* is a similarity scoring function over Σ_*g*_ if, for all *x*, *y* ∈ Σ, we have

(1) *p*(*x*, *x*) > 0,

(2) *p*(*x*, *x*) > *p*(*x*, *y*),

(3) *p*(*x*, *y*) = *p*(*y*, *x*),

(4) *p*(*x*, “−”) ≤ 0,

(5) *p*(“−”, “−”) = −∞.

Similarly, we say *p* is a distance scoring function over Σ_*g*_ if, for all *x*, *y*, *z* ∈ Σ, we have

(1) *p*(*x*, *x*) = 0,

(2) *p*(*x*, *y*) > 0,

(3) *p*(*x*, *y*) = *p*(*y*, *x*),

(4) *p*(*x*, “−”) > 0,

(5) *p*(*x*, *z*) ≤ *p*(*x*, *y*) + *p*(*y*, *z*),

(6) *p*(“−”, “−”) = *∞*.

##### Definition 2.3 (optimal global alignment)

Let Σ be an alphabet, Σ_*g*_ = Σ ∪ {“−”} be the extension of Σ with the gap character “−” and consider two strings *s* ∈ Σ^*n*^, *t* ∈ Σ^*m*^. Let *α*(*s*, *t*) = (*s*′, *t*′) be a valid global alignment of *s* and *t* (valid in the sense that it satisfies the conditions of Definition 2.1), 
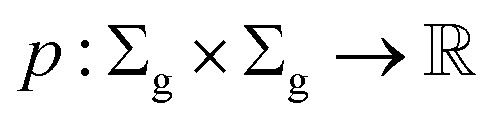
 be a scoring function over Σ_*g*_ and 
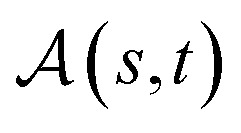
 be the space of all valid alignments of *s* and *t*. The score 
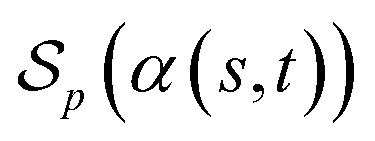
 of *α*(*s*, *t*) with respect to the scoring function *p* is defined as
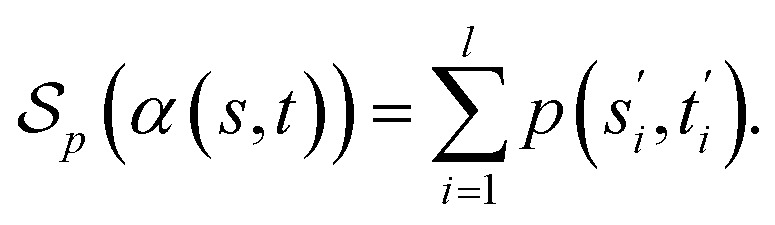
If *p* is a similarity scoring function, then the optimal global alignment α*(*s*, *t*) with respect to *p* is defined as
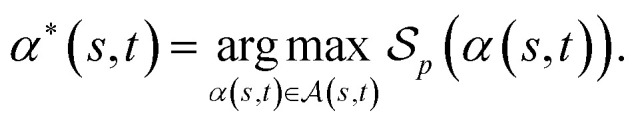
Similarly, if *p* is a distance scoring function then the optimal global alignment α*(*s*, *t*) with respect to *p* is defined as
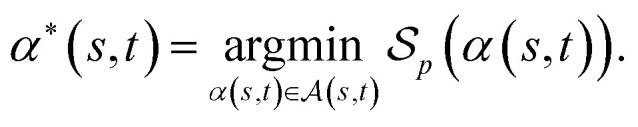


Since an alignment is optimal with respect to a given scoring function, it is natural to consider which scoring function to use in order to obtain the most meaningful alignments. In the context of biological sequences, researchers have been considering this problem for many years. A number of families of scoring matrices have been designed to encode useful notions of similarity. In this work, we only considered the BLOSUM62 scoring matrix,^46^ as it is a standard choice when performing sequence alignment.

#### 2.2.3 Levenshtein distance

The string edit distance defines a useful notion of distance between a pair of strings. It is informally defined as the minimum number of edit operations required to transform one string into another. The Levenshtein distance is a variant of the string edit distance that allows the operations of substitution, deletion and insertion of characters, and these are defined in Definition 2.4.

##### Definition 2.4 (elementary edit operations)

Let Σ be an alphabet. For two characters *a*, *b* ∈ Σ, we denote by *a* → *b* the elementary edit operation that substitutes *a* with *b*. Denoting by *ε* the null character (the empty string), we can define the elementary edit operations of insertion and deletion as ε → *b* and *a* → ε, respectively.

In order to define more complex transformations, one can consider the consecutive application of a sequence of elementary edit operations. Of special interest to the Levenshtein distance are those sequences of operations that transform one string into another. Such a sequence is known as an edit path and its length is defined as the number of operations in the sequence. The Levenshtein distance between two strings is defined as the length of the minimum length edit path, as seen in Definition 2.5.

##### Definition 2.5 (Levenshtein distance)

Let Σ be an alphabet and consider two strings *s* ∈ Σ^*n*^ and *t* ∈ Σ^*m*^ from Σ. An edit path from *s* to *t* is denoted by *P*_*s*,*t*_ and represents a sequence of elementary edit operations that transforms *s* into *t*. Denote by |*P*_*s*,*t*_| the number of operations contained in *P*_*s*,*t*_ and by 
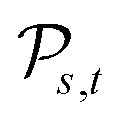
 the space of all edit paths from *s* to *t*. The Levenshtein distance *d*_L_(*s*, *t*) between *s* and *t* is defined as
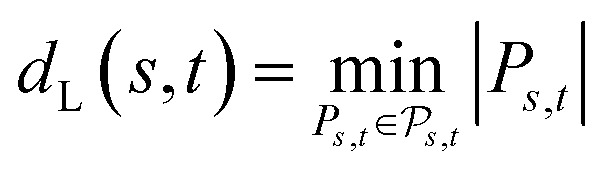


Definition 2.5 shares some interesting similarities with Definition 2.3. Both problems solve a combinatorial optimisation problem and, indeed, the Levenshtein distance can be realised as a special case of global alignment. More specifically, consider the distance scoring function *p*: Σ_*g*_ × Σ_*g*_ → {0, 1} defined as
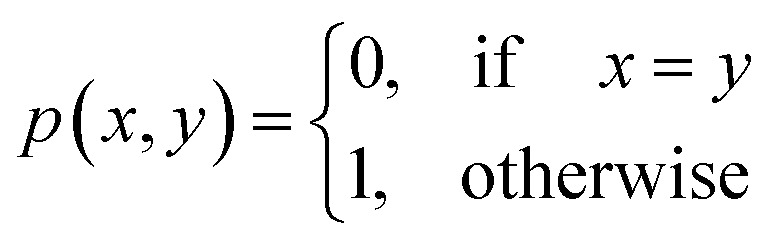


For two strings *s* ∈ Σ^*n*^ and *t* ∈ Σ^*m*^, let their optimal global alignment with respect to *p* be equal to α*(*s*, *t*) = {*s*′, *t*′}. Define the set *U* as
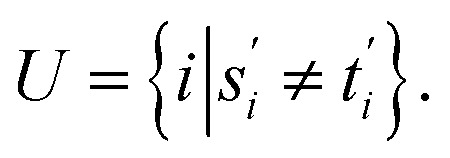


Then the score 
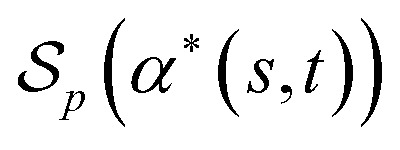
 of α*(*s*, *t*) with respect to *p* is equal to the cardinality of *U*. This is exactly equal to the Levenshtein distance between *s* and *t*.

#### 2.2.4 Local alignments

A global alignment produces an alignment which spans the whole length of a pair of strings. It is based on the assumption that the strings are related in their entirety. This assumption can be restrictive, since it is often the case that certain substrings exhibit high similarity whilst others do not. A local alignment produces an alignment that finds those high similarity substrings. That is, it finds the highest scoring global alignment from all possible substrings of the pair of strings. We formalise this notion in Definition 2.6.

##### Definition 2.6 (optimal local alignment)

Let Σ be an alphabet, Σ_*g*_ = Σ ∪ {“−”} be the extension of Σ with the gap character “−” and 
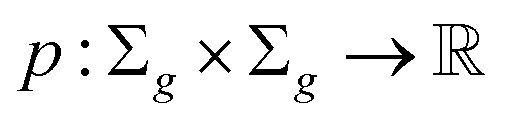
 be a similarity scoring function. For a string *s* ∈ Σ^*n*^, let 
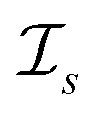
 be the space of all index sets such that for any 
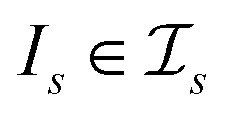
, *s*[*I*_*s*_] is a valid substring of *s*. Similarly, for a string *t* ∈ Σ^*m*^, let 
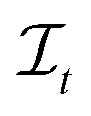
 be the space of all index sets such that for any 
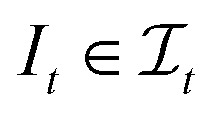
, *t*[*I*_*t*_] is a valid substring of *t*. For any 
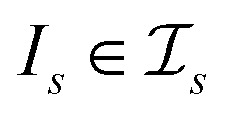
 and 
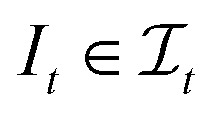
, denote by α*(*s*[*I*_*s*_], *s*[*I*_*t*_]) the optimal global alignment of *s*[*I*_*s*_] and *t*[*I*_*t*_] with respect to *p*. The optimal local alignment 
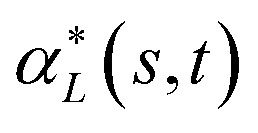
 of *s* and *t* with respect to *p* is defined as
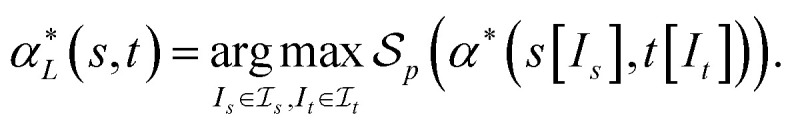


### 2.3 Computational methodology

This section discusses the setup of our computational evaluation, as well as the datasets used.

#### 2.3.1 Computational setup

To assess the usage of learning with sequence alignment functions, we performed a series of computational experiments on a number of AMP datasets. In each of our evaluations, we tested both the local alignment score (LA) and the Levenshtein distance (LEV) in conjunction with the Kreĭn-SVM algorithm. We compare against two baselines: an SVM with amino acid composition kernel and an SVM using the gapped *k*-mer kernel. The former is a positive-definite kernel function; peptides are represented *via* their amino acid composition and the kernel is defined as the inner product under this representation. The latter is also a positive-definite kernel function. It has produced accurate models in a number of biological-sequence classification tasks^47–49^ and hence makes for a useful baseline. When applicable, we also compared our models with AMP identification tools from the literature. The parasail package^50^ was used to compute local alignment scores. We only considered normalised variants of the local alignment score and Levenshtein distance, with the normalisation performed according to Schölkopf *et al.*^51^ and Yujian and Bo,^43^ respectively. We report the accuracy, the area under the receiver operating characteristic curve (AUC) and Matthews correlation coefficient (MCC) to compare models. The accuracy and MCC are defined in eqn (3), where TP, TN, FP and FN are the number of true-positives, true-negatives, false-positives and false-negatives, respectively.3
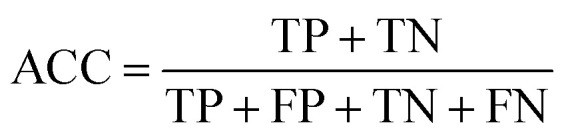
4
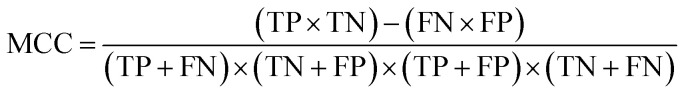


The AUC is defined as the probability that a classifier will score a randomly chosen positive instance higher than a randomly chosen negative instance.^52^ In order to allow for a fair comparison, all models used the same training and test splits. The optimal hyperparameters were selected by performing an exhaustive grid search over the training set, using 10-fold cross validation. The *λ* hyperparameter of the SVM algorithm, as well as the *λ*_+_ and *λ*_−_ hyperparameters of the Kreĭn-SVM algorithm, were selected from {0.01, 0.1, 1, 10, 100}. The Levenshtein distance and amino acid composition kernel have no hyperparameters to control; we used the default values for the hyperparameters of the local alignment score. The gapped *k*-mer kernel has two hyperparameters *g* and *m* and is quite susceptible to their values. The optimal value of *g* was selected from {1, 2, 3, 4, 5} and the optimal value of *m* was selected from {1, 2, 3, …, 10}. It is required that *g* > *m*, so only valid combinations of the two were considered. In our nested cross-validation experiments, we used 10 inner and 10 outer folds and the reported results are averaged over the outer fold test sets.

#### 2.3.2 General antimicrobial datasets

**Fig. 1:**
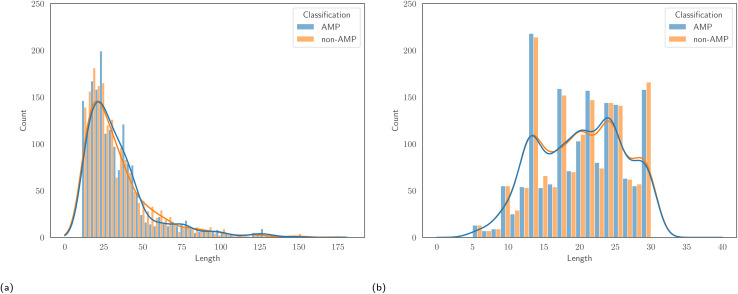
The distribution of peptide lengths for (a) the AMPScan and (b) DeepAMP datasets.

**Fig. 2:**
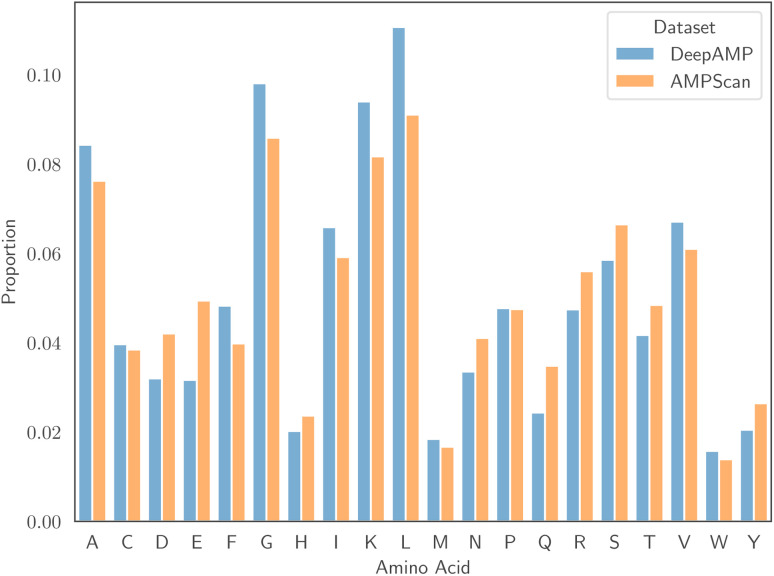
The distribution of amino acids for the AMPScan and DeepAMP datasets.

We selected two AMP classification datasets from the literature, which we refer to as AMPScan^26^ and DeepAMP,^25^ in order to test the ability of approach to predict general antimicrobial activity. Detailed discussions on the creation of these datasets can be found in the original studies. The AMPScan and DeepAMP datasets contain 3556 and 3246 instances, respectively. Each dataset also contains a 50 : 50 ratio of AMPs to non-AMPs, allowing us to avoid issues that result from class imbalance. Associated to each dataset is a specific test set, and reporting results on this set allows comparison with the authors' proposed models. Despite being of similar size, one major differentiating factor between the two datasets is the length of peptides. Fig. 1 displays the empirical distribution of peptide lengths for both datasets, partitioned by peptide classification. In both cases, the distributions corresponding to AMPs and non-AMPs are very similar. However, the distributions across datasets are clearly very different. The Kolmogorov–Smirnov two-sample test^53^ provides evidence to reject the null hypothesis that the peptide length distributions of the AMPScan and DeepAMP datasets are identical. DeepAMP contains generally shorter peptides than AMPScan. Indeed, the DeepAMP dataset was curated since short-length AMPs have been shown to exhibit enhanced activity, lower toxicity and higher stability as opposed to their longer counterparts.^54,55^ More importantly, synthesis is cheaper for the short AMPs than the full-length AMPs, which increases the potential for clinical translation and commercialisation.^12^Fig. 2 displays the empirical amino acid distributions for both datasets, indicating their similarity.

#### 2.3.3 Species-specific datasets

To test the ability of our methodology to identify activity against specific species, we have utilised an external dataset of 16 peptides with minimum inhibitory concentration (MIC) measured against *S. aureus* ATCC 29213 and *P. aeruginosa* ATCC 27853. To make this dataset suitable for classification, we label a peptide as active if its MIC <100 μg mL^−1^ and inactive otherwise. Further details of the microbiological experimentation used to construct this dataset can be found in Section 2.4.

Vishnepolsky *et al.*^56^ have shown that, given an appropriate training dataset, predictive models of peptide activity against specific species can be constructed. This involves training a separate model for each species of interest, which, in this case, was the DBSCAN algorithm.^57^ Their model predicting activity against *E. coli* ATCC 25922 achieved a balanced accuracy of 0.79, which was greater than a number of common AMP prediction tools.^56^ Furthermore, models to predict activity against *S. aureus* ATCC 25923 and *P. aeruginosa* ATCC 27853 were made publicly available as web-tools.

We follow the methodology of Vishnepolsky *et al.* to construct useful training datasets for our problem. We utilised the Database of Antimicrobial Activity and Structure of Peptides (DBAASP) as a source of data. DBAASP contains peptide activity measurements against a wide-range of species,^58^ including those of interest to us. We extracted from DBAASP all peptides with activity measured against *S. aureus* ATCC 29213, *S. aureus* ATCC 25923 or *P. aeruginosa* ATCC 27853 subject to the following conditions: (i) peptide length in the range [6, 18], (ii) without intrachain bonds, (iii) without non-standard amino acids and (iv) MIC measured in μg mL^−1^ or μM. Condition (i) was imposed as that is the range of peptide lengths in our external test set. Conditions (ii) and (iii) were imposed following the recommendation of the Vishnepolsky *et al.* and condition (iv) was imposed as conversion from μM to μg mL^−1^ is possible by estimating the molecular weight of a given sequence. Since no web-tool to predict activity against *S. aureus* ATCC 29213 was available, we couldn't directly compare our results. Instead, we collected data for peptides active against *S. aureus* ATCC 25923. This allowed us to compare our models with the state of the art provided by Vishnepolsky *et al.*

Three separate datasets of peptides with activity measured against *S. aureus* ATCC 29213, *S. aureus* ATCC 25923 and *P. aeruginosa* ATCC 27853 were created using the data collected from DBAASP. We refer to these datasets as SA_29213_, SA_25923_, and PA_27853_, respectively. The peptides in the respective datasets were labelled according to their activity against the specific strain. Each dataset is constructed from highly active peptides (MIC ≤25 μg mL^−1^) and inactive peptides (MIC ≥100 μg mL^−1^). A peptide with 25 μg mL^−1^ < MIC <100 μg mL^−1^ would not be included in our training dataset. This large interval allows us to account for experimental errors, which in turn increases the confidence in our class labels. In the case that a peptide was associated to multiple activity measurements, the median value was taken to represent its activity. As shown in Table 1, the three training datasets are all relatively small and contain slightly more active peptides than inactive peptides.

**Table 1:** Descriptive statistics of the SA_29213_, SA_25923_ and PA_27853_ datasets

Dataset	Size	Class ratio
SA_29213_	463	0.644
SA_25923_	808	0.646
PA_27853_	686	0.547

### 2.4 Microbiological experiments

A previously established dataset of 16 short-length peptides (18 amino acids or shorter in length) was used to test the ability of the developed ML algorithms in predicting antimicrobial activity against *S. aureus* and *P. aeruginosa*.^7^ These peptides were commercially synthesised by Mimotopes (Mulgrave Victoria, Australia) *via* solid phase Fmoc synthesis and were purified by reverse phase high performance liquid chromatography (RP-HPLC) to >95% purity. The efficacy of these peptides was already experimentally validated using established MIC assay with broth microdilution method approved by the Clinical and Laboratory Standards Institute. Full detail of the previously conducted microbiological experiment can be found in the previous study.^7^

## 3 Results

In Section 3.1, we discuss the ability of our models to identify general antimicrobial activity. We observe that our proposed models consistently outperform the baselines and, in some cases, the models proposed in the literature. One shortcoming of any computational model that identifies general antimicrobial activity is that it cannot be used to identify activity against specific species. We address this shortcoming in Section 3.2, by training our models to identify activity against *S. aureus* ATCC 29213 and *P. aeruginosa* ATCC 27853 on an experimentally validated dataset of 16 peptides, for which the proposed approach produces accurate models.

### 3.1 Identifying general antimicrobial activity

#### 3.1.1 Nested cross-validation

The performance of the AMP classifiers on the considered datasets is reported in Table 2. The results are averaged over the multiple test sets generated by nested-cross validation. On both datasets, the proposed models achieve a greater average accuracy, AUC and MCC than the baselines, with the local alignment score achieving the best values in all cases. The Welch *t*-test,^59^ with *p* = 0.05, is used to compare the test set AUC of our proposed models against the baseline SVM with Gapped *k*-mer kernel across the outer folds of nested cross-validation. Adjusting for the testing of multiple hypotheses with the Bonferroni correction, we observe a significant difference between the mean AUC of the local alignment score and that of the baseline SVM with Gapped *k*-mer kernel on the AMPScan dataset. All other comparisons, including those on the DeepAMP dataset, are not significant. The performance of all models is greater on the AMPScan dataset than on the DeepAMP dataset. This may, in part, be due to the fact that DeepAMP contains generally shorter peptides. Shorter peptides provide less information to the models, which can limit their discriminative ability.

**Table 2:** Quality of the predictions on the AMPScan and DeepAMP datasets. The average accuracy AUC and MCC are reported (standard deviation in parentheses), computed over the outer fold test sets of the nested cross-validation procedure. Results are presented for the Kreĭn-SVM with local alignment score (LA-KSVM), the Kreĭn-SVM with Levenshtein distance (LEV-KSVM), the SVM with Gapped *k*-mer kernel (GKM-SVM) and the SVM with amino acid composition kernel (AAC-SVM)

Model	AMPScan			DeepAMP		
	Accuracy	AUC	MCC	Accuracy	AUC	MCC
LA-KSVM	0.920 (0.017)	0.969 (0.006)	0.842 (0.033)	0.760 (0.025)	0.821 (0.028)	0.522 (0.051)
LEV-KSVM	0.910 (0.021)	0.966 (0.010)	0.821 (0.042)	0.756 (0.032)	0.819 (0.029)	0.513 (0.063)
GKM-SVM	0.899 (0.015)	0.957 (0.007)	0.799 (0.030)	0.751 (0.032)	0.817 (0.029)	0.506 (0.066)
AAC-SVM	0.865 (0.023)	0.930 (0.009)	0.732 (0.044)	0.723 (0.031)	0.784 (0.035)	0.447 (0.061)

#### 3.1.2 Predefined test set

Table 3 reports the results of all models on the predefined test set associated with each dataset. For the sake of completeness, we also include the performance of the neural network-based classifiers proposed by the authors of each dataset. As for the nested cross-validation results, we observe that all models perform better on the AMPScan dataset than the DeepAMP dataset. Considering the former, the local alignment score achieves the largest accuracy, AUC and MCC, and is followed closely by the literature model. On the DeepAMP dataset, the performance is similar among all methods. The local alignment score achieves the best AUC but the Levenshtein distance achieves the best accuracy and MCC. However, the Levenshtein distance outperforms the literature model against all metrics. On both datasets, the baselines are the least predictive models. It is encouraging to observe that the sequence alignment functions can produce classifiers that match, and also outperform, the neural network-based classifiers.

**Table 3:** Quality of the predictions on the AMPScan and DeepAMP datasets. The accuracy, AUC and MCC are reported, computed on the predefined test sets. Results are presented for the Kreĭn-SVM with local alignment score (LA-KSVM), the Kreĭn-SVM with Levenshtein distance (LEV-KSVM), the SVM with Gapped *k*-mer kernel (GKM-SVM) and the SVM with amino acid composition kernel (AAC-SVM). Results from the respective neural network-based classifiers^25,26^ proposed by the authors of each dataset are also presented, denoted by Literature

Model	AMPScan			DeepAMP		
	Accuracy	AUC	MCC	Accuracy	AUC	MCC
LA-KSVM	0.911	0.967	0.823	0.761	0.863	0.523
LEV-KSVM	0.904	0.960	0.809	0.798	0.860	0.596
GKM-SVM	0.900	0.954	0.801	0.782	0.838	0.564
AAC-SVM	0.870	0.929	0.742	0.771	0.853	0.543
Literature	0.910	0.965	0.820	0.771	0.853	0.543

### 3.2 Identifying species-specific activity

In this section, we highlight the ability of our models to identify AMPs that are active against specific species, particularly *S. aureus* ATCC 29213 and *P. aeruginosa* ATCC 27853. Table 4 displays the accuracy on the external test set for models trained on the AMPScan and DeepAMP datasets. Once again, we also include the performance of the neural-network-based classifiers proposed by the authors of the AMPScan and DeepAMP datasets. We observe that the performance of all models is very poor. We noticed in our investigations that the majority of models predicted active for a large proportion of the peptides. This general poor performance is to be expected. We attribute it to the fact that these models have been trained to recognise if a peptide exhibits antimicrobial activity against any type of species. It is therefore unreasonable to assume that they are able to discriminate activity against a specific species.

**Table 4:** Predictive accuracy of the Kreĭn-SVM with local alignment score (LA-KSVM), the Kreĭn-SVM with Levenshtein distance (LEV-KSVM) and the SVM with Gapped *k*-mer kernel (GKM-SVM) on the species-specific test sets of 16 peptides. Results from the respective neural network-based classifiers^25,26^ proposed by the authors of each dataset are also presented, denoted by literature. The dataset column indicates which dataset a model was trained on. The heading *S. aureus* indicates the model was predicting activity against *S. aureus* ATCC 29213 and the heading *P. aeruginosa* indicates the model was predicting activity against *P. aeruginosa* ATCC 27853

Model	Dataset	*S. aureus*	*P. aeruginosa*
LA-KSVM	AMPScan	0.312	0.312
	DeepAMP	0.250	0.250
LEV-KSVM	AMPScan	0.312	0.312
	DeepAMP	0.312	0.312
GKM-SVM	AMPScan	0.312	0.312
	DeepAMP	0.375	0.375
AAC-SVM	AMPScan	0.312	0.312
	DeepAMP	0.312	0.312
Literature	AMPScan	0.250	0.250
	DeepAMP	0.438	0.438

Table 5 displays the accuracy on the external test set for models trained on the species-specific datasets. We also present the performance of the web-tools provided by Vishnepolsky *et al.*^56^ As mentioned in Section 2.3.3, at the time of publication, there is no web-tool to predict activity against *S. aureus* ATCC 29213. Hence, we also provide results for models trained on SA_25923_ and tasked with predicting activity against *S. aureus* ATCC 29213. There is clearly a general improvement over the models trained on the AMPScan and DeepAMP datasets, indicating that the models have much greater discriminative power. On the SA_29213_ dataset, the baseline SVM with amino acid composition kernel is the most predictive with respect to all the metrics. All remaining models achieve the same accuracy and MCC. Considering the SA_25923_ dataset, the Levenshtein distance produces a model with the same accuracy and MCC as the web-tool but with a larger AUC. It also achieves the same accuracy and AUC as the baseline SVM with amino acid composition kernel, but with a larger MCC. It is interesting to note that the models trained on SA_25923_ can still make accurate predictions on *S. aureus* ATCC 29213. Whilst these are two different strains, the findings suggest that the antimicrobial susceptibility to the AMPs is similar for both strains, implying similar mechanisms work in the same species. Considering the PA_27853_ dataset, the baseline SVM with amino acid composition kernel performs the best against all metrics. We find that the local alignment score, Levenshtein distance and baseline SVM with Gapped *k*-mer kernel produce equally accurate models, all of which are more accurate than the web-tool. However, the AUC of the local alignment score and Levenshtein distance are considerably higher than that of the baseline SVM with Gapped *k*-mer kernel. Whilst it is difficult to make any strong conclusions on such a small dataset, it is still encouraging to observe that our models achieve similar accuracy to both the baseline models and web-tools.

**Table 5:** Quality of the predictions of the Kreĭn-SVM with local alignment score (LA-KSVM), the Kreĭn-SVM with Levenshtein distance (LEV-KSVM), the SVM with Gapped *k*-mer kernel (GKM-SVM) and the SVM with amino acid composition kernel (AAC-SVM) on the test sets of 16 peptides. Results from the DBAASP Web-tools are also presented.^56,58^ The headings in the third to fifth columns indicate which dataset the models were trained on. The models trained on both SA_29213_ and SA_25923_ were tasked with predicting activity against *S. aureus* ATCC 29213. The models trained on PA_27853_ were tasked with predicting activity against *P. aeruginosa* ATCC 27853. Numbers highlighted in bold indicate the largest AUC achieved on the respective dataset

Model	Evaluation metric	Training dataset		
		SA_29213_	SA_25923_	PA_27853_
LA-KSVM	Accuracy	0.688	0.688	0.750
	AUC	0.873	0.909	0.891
	MCC	0.522	0.405	0.595
LEV-KSVM	Accuracy	0.688	0.875	0.750
	AUC	0.927	**0.982**	0.855
	MCC	0.522	0.764	0.595
GKM-SVM	Accuracy	0.688	0.688	0.750
	AUC	0.945	0.891	0.655
	MCC	0.522	0.405	0.389
AAC-SVM	Accuracy	0.875	0.875	0.875
	AUC	**0.964**	**0.982**	**0.964**
	MCC	0.709	0.709	0.764
DBAASP Web-tool	Accuracy	—	0.875	0.688
	AUC	—	0.945	0.718
	MCC	—	0.764	0.522

## 4 Conclusions

We have assessed the capabilities of sequence alignment functions coupled with the Kreĭn-SVM as AMP classification models. Our investigations indicate that the proposed methodology produces accurate classifiers of both general and species-specific antimicrobial activity of AMPs. The utility of our methodology is twofold. Firstly, since sequence alignment algorithms operate directly on amino acid sequences, these methods do not explicitly require the use of peptide features. This removes the need for the practitioner to decide which features to use, which is often a detailed and time-consuming process. Secondly, in all of our experiments, we used the local alignment score with its default hyperparameters. Having achieved such promising results, it prompts the question of whether more accurate models could be attained by also tuning the various hyperparameters of the local alignment score, such as the choice of scoring function, which we will explore in future work.

As the chemical space of natural peptides is extremely large, the development of highly accurate classifiers will help accelerate the discovery and development of novel *de novo* AMPs. In addition, the promising results generated from this study open a number of possible avenues for further work. Our identification of activity against specific species could be improved using a larger external test set, allowing us to draw stronger conclusions. Furthermore, the methodology we have presented is not specific to the classification of AMPs. We suspect that the Kreĭn-SVM coupled with sequence alignment functions could be applied to other biological-sequence classification tasks. Our computational findings demonstrate not only the feasibility of the proposed approach but more generally the utility of the Kreĭn-SVM as a classification algorithm. Its use of indefinite kernel functions provides a means for practitioners to learn from domain-specific similarity functions without the concern of verifying the positive-definite assumption. This is beneficial since it is often well beyond the expertise of the practitioner to verify this assumption. Whilst we have explored its use when combined with sequence alignment functions, there exist many more indefinite kernel functions with which the Kreĭn-SVM could be combined. Furthermore, the theoretical insight of separately regularising the decomposition components of a function in a Kreĭn space could be applied to develop other indefinite kernel-based learning algorithms. A notable example that relates to the current study would be to extend the One-Class SVM^60^ to incorporate indefinite kernel functions. The One-Class SVM is a kernel-based learning algorithm that performs anomaly detection.^61^ It has previously been applied to identify the domain of applicability of virtual screening models.^62^ An indefinite kernel extension of the One-Class SVM could be directly applied to estimate the domain of applicability of our models.

## Data availability

The source code and data used to reproduce the computational experiments is available at https://github.com/Mrjoeybux/KreinAMP.

## Author contributions

Joseph Redshaw: conceptualisation, data curation, formal analysis, investigation, software, methodology, visualisation, writing – original draft. Darren S. J. Ting: conceptualisation, methodology, resources, writing – original draft. Alex Brown: validation, software, writing – review & editing. Jonathan D. Hirst: conceptualisation, methodology, project administration, resources, supervision, writing – review & editing. Thomas Gärtner: conceptualisation, methodology, supervision, writing – review & editing.

## Conflicts of interest

A. B. is an employee and shareholder in GSK.

## Supplementary Material
